# Health-related quality of life in patients with giant cell arteritis treated with tocilizumab in a phase 3 randomised controlled trial

**DOI:** 10.1186/s13075-019-1837-7

**Published:** 2019-02-20

**Authors:** Vibeke Strand, Sophie Dimonaco, Katie Tuckwell, Micki Klearman, Neil Collinson, John H. Stone

**Affiliations:** 1 Division of Immunology/Rheumatology, Stanford University, Palo Alto, CA USA; 2 Roche Products Ltd., Welwyn Garden City, UK; 3 Genentech, South San Francisco, CA USA; 4 Massachusetts General Hospital Rheumatology Unit, Harvard Medical School, Yawkey 2, 55 Fruit Street, Boston, MA 02114 USA

**Keywords:** DMARDs (biologic), Inflammation, Giant cell arteritis, Patient-reported outcomes, Quality of life, Tocilizumab

## Abstract

**Background:**

Patients with giant cell arteritis (GCA) treated with tocilizumab (TCZ) every week or every other week and prednisone tapering achieved superior rates of sustained remission to patients treated with placebo and prednisone tapering in a randomised controlled trial. Health-related quality of life (HRQOL) in patients from this trial is now reported.

**Methods:**

Exploratory analyses of SF-36 PCS and MCS and domain scores, PtGA and FACIT-Fatigue were performed in patients treated with weekly subcutaneous TCZ 162 mg plus 26-week prednisone taper (TCZ-QW + Pred-26) or placebo plus 26-week or 52-week prednisone tapers (PBO + Pred-26 or PBO + Pred-52). These analyses were performed on responder and non-responder patients, including those who achieved the primary outcome and those who experienced flare and received escape prednisone doses.

**Results:**

Baseline SF-36 PCS, MCS and domain scores were low, indicating impaired HRQOL related to GCA. At week 52, least squares mean (LSM) changes in PCS scores improved with TCZ-QW + Pred-26 but worsened in both PBO + Pred groups (*p* <  0.001). LSM changes in MCS scores increased with TCZ-QW + Pred-26 versus PBO + Pred-52 (*p* < 0.001). Treatment with TCZ-QW + Pred-26 resulted in significantly greater improvement in four of eight SF-36 domains compared with PBO + Pred-26 and six of eight domains compared with PBO + Pred-52 (*p* < 0.01). Improvement with TCZ-QW + Pred-26 met or exceeded minimum clinically important differences (MCID) in all eight domains compared with five domains with PBO + Pred-26 and none with PBO + Pred-52. Domain scores in the TCZ-QW + Pred-26 group at week 52 met or exceeded age- and gender-matched normative values (A/G norms). LSM changes from baseline in FACIT-Fatigue scores increased significantly with TCZ-QW + Pred-26, exceeding MCID and A/G norms (*p* < 0.001).

**Conclusions:**

Patients with GCA receiving TCZ-QW + Pred-26 reported statistically significant and clinically meaningful improvement in SF-36 and FACIT-Fatigue scores compared with those receiving prednisone only. Improvements in the TCZ-QW + Pred-26 group led to recovery of HRQOL to levels at least comparable to those of A/G-matched normative values at week 52 and exceeded normative values in five of eight domains.

**Trial registration:**

ClinicalTrials.gov, NCT01791153. Date of registration: February 13, 2013.

## Background

Giant cell arteritis (GCA) is a systemic vasculitis that primarily affects medium- to large-sized arteries including the aorta, its primary branches, and vessels supplying the eyes and brain. The prevalence of GCA in the general population ranges from 0.01 to 0.25% [1–4], and it is estimated that this disease will be diagnosed in more than 3 million people in Europe, North America and Oceania by 2050 [5]. Glucocorticoid therapy, regarded for decades as the standard of care for GCA, is associated with a legion of potentially serious adverse effects and is often poorly tolerated [6]. Although it is generally recommended that glucocorticoid doses be tapered as rapidly as possible, many patients with GCA require unacceptably high doses over the long-term to control their symptoms and to prevent disease complications [7–9].

Health-related quality of life (HRQOL) has not been investigated extensively in patients with GCA and has not been reported in patients with GCA receiving treatment in a randomised controlled trial (RCT). Complications of GCA and adverse effects of long-term glucocorticoid treatment can result in physical and psychological problems that have the potential to impair HRQOL in patients with GCA [10]. However, reports on HRQOL assessment in patients with GCA are limited. Fears surrounding potential vision loss and dissatisfaction with the adverse effects of glucocorticoids were identified as the most important HRQOL concerns in a survey of patients with GCA [11], but vision loss was not associated with impaired HRQOL at baseline or after treatment with glucocorticoids with or without methotrexate in an RCT that enrolled too few patients for a treatment effect to be investigated [12]. In a case-control study, HRQOL was not impaired in patients with GCA either before or after glucocorticoid treatment compared with controls [13], though this study examined only HRQOL data after high-dose glucocorticoid treatment had been discontinued (the 30 patients in the analysis had either stopped corticosteroid treatment or were taking long-term low doses of glucocorticoids).

GiACTA was a 52-week phase 3 RCT that led to the approval of tocilizumab (TCZ) for the treatment of GCA in the USA [14], the European Union [15] and other countries. We report here patient-reported outcomes (PROs) from this trial, including comparison between the treatment groups for 36-Item Short-Form Health Survey (SF-36) Physical Component Summary (PCS) and Mental Component Summary (MCS) scores and domains, Functional Assessment of Chronic Illness Therapy (FACIT)-Fatigue and Patient Global Assessment of Disease Activity (PtGA). We also compare reported improvements at 52 weeks among treatment groups that are greater than minimum clinically important differences (MCIDs) or age- and gender-matched normative values (A/G norms). This is the first time that HRQOL has been reported in patients with GCA treated in an RCT, and, as such, the findings provide important information on QOL improvements in patients treated with TCZ and tapered glucocorticoids.

## Patients and methods

### Patients

The GiACTA RCT (ClinicalTrials.gov, NCT01791153) design and enrolment criteria have been published [16, 17]. Briefly, patients ≥ 50 years of age with newly diagnosed or relapsing active GCA confirmed by temporal artery biopsy or cross-sectional imaging and a history of elevated erythrocyte sedimentation rate attributable to GCA were randomly assigned (2:1:1:1) to one of four 52-week regimens: (1) weekly subcutaneous TCZ 162 mg plus a 26-week prednisone taper (TCZ-QW + Pred-26), (2) every-other-week subcutaneous TCZ 162 mg plus a 26-week prednisone taper (TCZ-Q2W + Pred-26), (3) weekly subcutaneous placebo plus a 26-week prednisone taper (PBO + Pred-26) or (4) placebo plus a 52-week prednisone taper (PBO + Pred-52). The trial was conducted in accordance with Good Clinical Practice guidelines and the Declaration of Helsinki. All patients provided written informed consent as approved by the institutional review board or ethics committee. The primary outcome (sustained disease remission to week 52) and secondary and exploratory PRO outcomes (SF-36, FACIT-Fatigue and PtGA) were analysed at 1 year.

### Patient-reported outcome assessments

We analysed data by SF-36 PCS and MCS and all eight individual domains. These domains are physical function, role physical, bodily pain, general health, vitality, social function, role emotional and mental health. We also analysed PtGA by visual analogue scale (VAS) and FACIT-Fatigue score. We used MCIDs previously validated in patients with rheumatoid arthritis and systemic lupus erythematosus because such values have never been defined in GCA.

Least square mean (LSM) changes from baseline to week 52 in SF-36 PCS and MCS scores were assessed using a range of 0 to 50, with normative scores of 50 and a standard deviation of ± 10; higher scores indicate better HRQOL [18]. Change ≥ 2.5 in PCS and MCS was considered the MCID [19]. SF-36 domain scores ranging from 0 to 100 were compared with A/G norms matched to the study population. LSM changes from baseline to week 52 were depicted using spydergrams [20]. An MCID of 5.0 was used for changes in domain scores [19]. Overall changes in SF-36 scores in spydergrams were quantified using the SF-6D utility score based on mean scores across all eight domains [21, 22] and the previously defined minimally important difference (MID) for an SF-6D utility score of 0.041 [23]. Changes from baseline to week 52 in PtGA, ranging from 0 to 100, were assessed by VAS using the generally accepted MCID of 10.0 [19]. FACIT-Fatigue was assessed on a scale of 0 to 52, with higher scores representing less fatigue, using an MCID of 4.0 [24].

### Statistical analysis

The current analyses compared PROs between the TCZ-QW + Pred-26 group and the PBO + Pred-26 and PBO + Pred-52 groups. These comparisons were selected to correspond with the primary and key secondary analyses of sustained remission from baseline to week 52 in the GiACTA trial [16]. Comparisons of SF-36 PCS and MCS scores between the TCZ-Q2W + Pred-26 group and the two PBO + Pred groups were not statistically significant at the pre-specified 1% level. Therefore, data from that group were not assessed further. Our analyses focused on the TCZ-QW + Pred-26 group, the PBO + Pred-26 group and the PBO + Pred-52 group.

All patients in the intent-to-treat (ITT) population, including those who experienced disease flare, were included in the current analyses. This approach contrasts with the analytical approach used previously, in which data obtained after use of escape therapy were censored [16]. Statistical significance was set as *p* < 0.01. All analyses presented were performed after unblinding and are considered exploratory.

## Results

### Patients

One hundred patients were randomly assigned to TCZ-QW + Pred-26, 50 to TCZ-Q2W + Pred-26, 50 to PBO + Pred-26 and 51 to PBO + Pred-52. One patient assigned to the TCZ-Q2W group did not receive any study treatment and was not included in the ITT population. Baseline demographics and disease characteristics for all patients have been reported [16, 25] and are shown for the TCZ-QW + Pred-26, PBO + Pred-26 and PBO + Pred-52 groups in Table 1. In the ITT population, patients in both PBO + Pred groups received approximately twice the cumulative dose of prednisone as patients receiving TCZ-QW + Pred-26 over 52 weeks. The median (minimum–maximum) cumulative prednisone doses were 1862.0 mg (630.0–6602.5) for TCZ-QW + Pred-26, 3296.0 mg (932.0–9777.5) for PBO + Pred-26 and 3817.5 mg (822.5–10,697.5) for PBO + Pred-52 (*p* < 0.001 for comparisons). Among patients in the escape population who remained in the study until week 52, the median (minimum–maximum) cumulative prednisone doses over 52 weeks were 3129.8 mg (2009.0–5680.5, *n* = 18) for TCZ-QW + Pred-26, 4023.5 mg (2583.5–8695.0, *n* = 29) for PBO + Pred-26 and 5389.5 mg (2700–10,697.5, *n* = 24) for PBO + Pred-52. In summary, in the ITT and escape populations, cumulative prednisone doses were far higher in both PBO + Pred groups than in the TCZ-QW + Pred-26 group.

**Table 1:** Baseline demographics and disease characteristics for the treatment groups assessed (all patients)

	TCZ-QW + Pred-26 *N* = 100	PBO + Pred-26 *N* = 50	PBO + Pred-52 *N* = 51
Age, years	69.5 (8.5)	69.3 (8.1)	67.8 (7.7)
Female, *n* (%)	78 (78.0)	38 (76.0)	37 (72.5)
Race, *n* (%)			
White	97 (97.0)	50 (100.0)	49 (96.1)
Black or African American	1 (1.0)	0	2 (3.9)
Asian	0	0	0
Other	1 (1.0)	0	0
Unknown	1 (1.0)	0	0
Weight, kg	69.8 (13.8)	70.1 (15.8)	73.1 (15.3)
Disease duration, days	306.8 (563.5)	364.7 (569.9)	255.2 (435.5)
Disease onset, *n* (%)			
Newly diagnosed	47 (47.0)	23 (46.0)	23 (45.1)
Relapsing	53 (53.0)	27 (54.0)	28 (54.9)
Prednisone dose, mg/day	34.6 (13.4)	34.6 (13.0)	34.5 (14.2)
Cranial signs or symptoms, *n* (%)	41 (41.0)	20 (40.0)	16 (31.4)
PMR symptoms	59 (59.0)	30 (60.0)	35 (68.6)
ESR, mm/h	24.6 (18.7)	28.8 (25.4)	24.2 (18.2)
SF-36 PCS	43.1 (9.4)	42.6 (10.9)	41.1 (10.0)
SF-36 MCS	42.8 (12.4)	42.7 (12.1)	40.5 (13.7)
FACIT-Fatigue	36.1 (11.1)	35.0 (12.8)	31.4 (13.6)
PtGA, 100 mm VAS	43.6 (25.7)	35.7 (28.2)	47.8 (27.8)

Data are mean (SD) unless stated otherwise

*ESR* erythrocyte sedimentation rate, *FACIT* Functional Assessment of Chronic Illness Therapy, *MCS* Mental Component Summary, *PBO* placebo, *PCS* Physical Component Summary, *PMR* polymyalgia rheumatica, *Pred-26* 26-week prednisone taper, *Pred-52* 52-week prednisone taper, *PtGA* Patient Global Assessment of Disease Activity, *QW* every week, *SD* standard deviation, *SF-36* 36-Item Short-Form Health Survey, *TCZ* tocilizumab, *VAS* visual analogue scale

### SF-36

At baseline, PCS and MCS scores were similar across treatment groups and approximately 1 standard deviation (SD) below the A/G-matched normative scores of 50. Patients in the TCZ-QW + Pred-26 group reported greater LSM improvements from baseline to week 52 in PCS scores than patients in both PBO + Pred groups (*p* < 0.01). LSM improvement in MCS was significant in the TCZ-QW + Pred-26 group compared with the PBO + Pred-52 group (*p* < 0.01). Changes from baseline exceeded the MCID in both PCS and MCS scores in the TCZ-QW + Pred-26 group. Change from baseline in MCS score to week 52 exceeded the MCID in the PBO + Pred-26 group (Table 2).

**Table 2:** Mean SF-36 PCS and MCS scores at baseline and week 52 (ITT population)

	SF-36 PCS			SF-36 MCS		
	TCZ-QW + Pred-26 *N* = 100	PBO + Pred-26 *N* = 50	PBO + Pred-52 *N* = 51	TCZ-QW + Pred-26 *N* = 100	PBO + Pred-26 *N* = 50	PBO + Pred-52 *N* = 51
Baseline	43.10 *n* = 97	42.65 *n* = 48	41.12 *n* = 49	42.77 *n* = 97	42.73 *n* = 48	40.45 *n* = 49
Week 52	47.75 *n* = 85	41.52 *n* = 43	41.24 *n* = 45	51.64 *n* = 85	49.36 *n* = 43	44.86 *n* = 45
LSM change from baseline to week 52	4.18*† *n* = 95	− 0.98 *n* = 47	− 0.40 *n* = 49	8.10†‡ *n* = 95	5.25† *n* = 47	1.89 *n* = 49

Data from patients who received prednisone escape therapy are included

*ITT* intent-to-treat, *LSM* least squares mean, *MCID* minimal clinically important difference, *MCS* Mental Component Summary, *PBO* placebo, *PCS* Physical Component Summary, *QW* every week, *SF-36* 36-Item Short-Form Health Survey, *TCZ* tocilizumab

*p < 0.001 vs PBO + Pred-26- and vs PBO + Pred-52

†Score ≥ MCID of 2.5 in patients with RA

‡*p* = 0.0727 vs PBO + 26-week taper and *p* < 0.001 vs PBO + 52-week taper

**Fig. 1:**
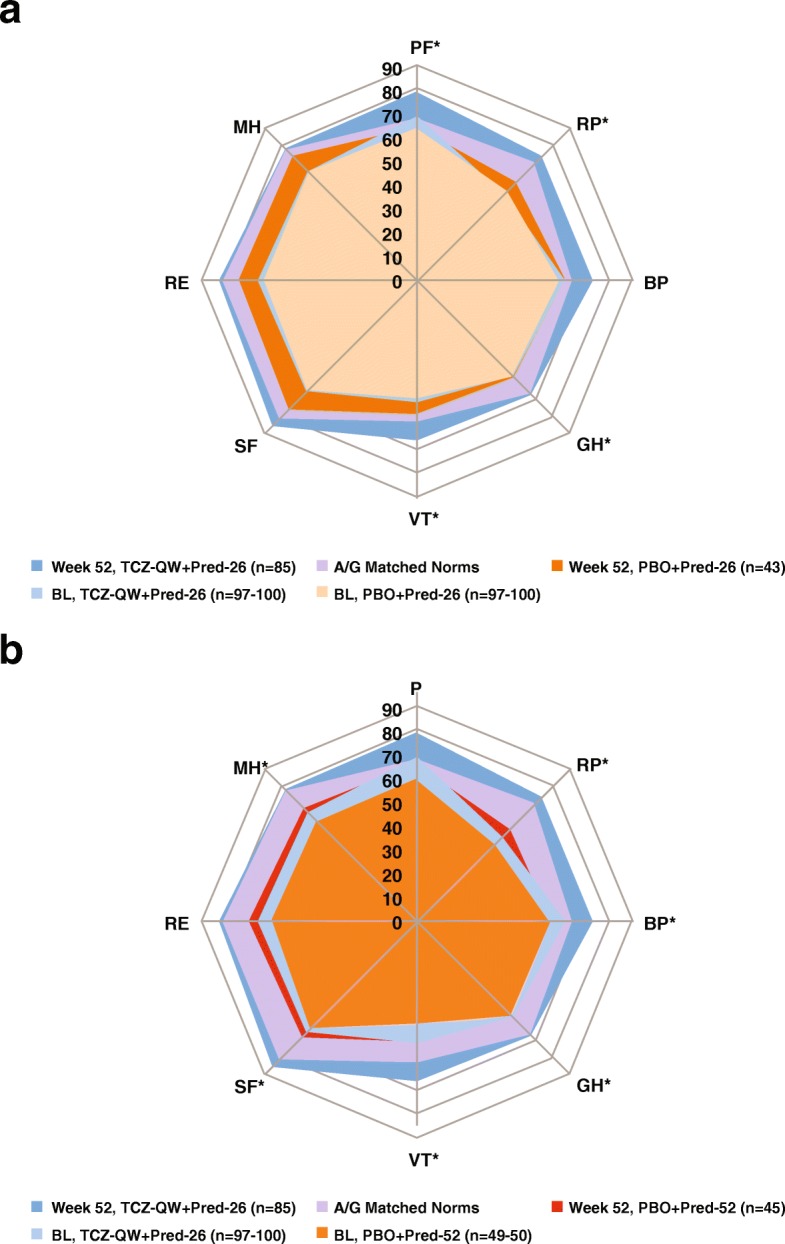
SF-36 domain scores at baseline and week 52. SF-36 domain scores are shown for the TCZ-weekly group compared with the placebo-26-week taper (**a**) and compared with the placebo-52-week taper (**b**) groups. Values shown in parentheses are the numbers of patients. *p < 0.01 TCZ-QW + Pred-26 vs PBO + Pred group. *A/G* age/gender, *BL* baseline, *BP* bodily pain, *GH* general health, *MH* mental health, *PBO* placebo, *PF* physical function, *Pred-26* 26-week prednisone taper, *Pred-52* 52-week prednisone taper, *QW* every week, *RE* role emotional, *RP* role physical, *SF* social function, *SF-36* 36-Item Short-Form Health Survey, *TCZ* tocilizumab, *VT* vitality

Baseline domain scores reflected impaired HRQOL, which was the most impacted in the role physical, vitality, social function, role emotional and mental health domains. Scores were lowest in the PBO + Pred 52 group, reflecting decrements of 16–25 points below A/G norms (Table 3, Fig. 1). At week 52, LSM changes from baseline with TCZ-QW + Pred-26 treatment exceeded those in the PBO + Pred-26 group in four domains (physical function, role physical, general health and vitality; *p* < 0.01 for all) and exceeded those in the PBO + Pred-52 group in six domains (role physical, bodily pain, general health, vitality, social function and mental health; *p* < 0.01 for all) (Table 3, Fig. 1). Reported improvements across all domains exceeded the MCID in the TCZ-QW + Pred-26 group compared with five of eight domains in the PBO + Pred-26 group and none in the PBO + Pred-52 group (Table 3).

**Table 3:** SF-36 domain scores and SF-6D utility scores (ITT population)

	A/G norms	TCZ-QW + Pred-26 *N* = 100			PBO + Pred-26 *N* = 50				PBO + Pred-52 *N* = 51			
								*p* value				*p* value
		BL	Wk 52	LSM Δ BL–Wk 52	BL	Wk 52	LSM Δ BL–Wk 52	TCZ-QW + Pred-26 vs PBO + Pred-26	BL	Wk 52	LSM Δ BL–Wk 52	*p*TCZ-QW + Pred-26 vs PBO + Pred-52
PF	67.56	69.10 *n* = 100	78.28 *n* = 85	*6.83* *n* = 98	63.60 *n* = 50	63.48 *n* = 43	− 3.43 *n* = 49	0.0024	59.40 *n* = 50	65.44 *n* = 45	2.68 *n* = 50	NS
RP	69.44	49.56 *n* = 100	73.75 *n* = 85	*20.64* *n* = 98	52.42 *n* = 49	58.28 *n* = 43	*5.87* *n* = 48	< 0.001	45.38 *n* = 50	53.89 *n* = 45	4.46 *n* = 50	< 0.001
BP	64.52	61.93 *n* = 100	73.25 *n* = 85	*10.89* *n* = 98	58.90 *n* = 50	61.77 *n* = 43	1.00 *n* = 49	NS	55.67 *n* = 49	56.27 *n* = 45	− 2.87 *n* = 49	< 0.001
GH	66.49	55.00 *n* = 97	65.81 *n* = 85	*9.06* *n* = 95	56.43 *n* = 49	57.28 *n* = 43	− 1.34 *n* = 48	< 0.001	55.69 *n* = 50	52.29 *n* = 45	− 4.05 *n* = 50	< 0.001
VT	58.65	50.19 *n* = 99	66.13 *n* = 85	*15.69* *n* = 97	49.00 *n* = 50	55.67 *n* = 43	*5.26* *n* = 49	0.0011	42.38 *n* = 50	49.17 *n* = 45	3.53 *n* = 50	< 0.001
SF	81.49	64.25 *n* = 100	84.71 *n* = 85	*17.35* *n* = 98	65.25 *n* = 50	75.87 *n* = 43	*9.83* *n* = 49	NS	63.00 *n* = 50	67.50 *n* = 45	2.34 *n* = 50	< 0.001
RE	82.08	66.38 *n* = 100	82.45 *n* = 85	*13.37* *n* = 98	64.63 *n* = 49	74.81 *n* = 43	*6.44* *n* = 48	NS	60.33 *n* = 50	69.63 *n* = 45	3.53 *n* = 50	NS
MH	77.16	64.04 *n* = 99	77.94 *n* = 85	*12.54* *n* = 97	64.20 *n* = 50	73.60 *n* = 43	*7.43* *n* = 49	NS	59.10 *n* = 50	66.33 *n* = 45	3.13 *n* = 50	< 0.001
SF-6D utility score	0.746	0.694	0.775	*0.080*	0.686	0.721	0.034	–	0.663	0.692	0.029	–

Changes meeting or exceeding the MCID of 5.0 for domain scores and meeting or exceeding the MID of 0.041 for SF-6D scores (based on patients with RA and SLE) are shown in italics

*A/G* age/gender, *BL* baseline, *BP* bodily pain, *GH* general health, *ITT* intent-to-treat, *LSM* least squares mean, *MH* mental health, *NS* not significant (*p* > 0.01), *PBO* placebo, *PF*, physical function, *QW* every week, *RE* role emotional, *RP* role physical, *SF* social function, *SF-36* 36-Item Short-Form Health Survey, *SLE* systemic lupus erythematosus, *TCZ* tocilizumab, *VT* vitality, *wk* week

### SF-6D utility scores

The SF-6D utility score (a quantitative measure of SF-36) was low in each treatment group at baseline, reflecting impaired HRQOL. Changes in SF-6D utility scores from baseline to week 52 exceeded the MID of 0.041 in the TCZ-QW + Pred-26 group (0.080) but in neither of the two PBO + Pred groups (0.034 for PBO + Pred-26 and 0.029 for PBO + Pred-52) (Table 3).

### Patient global assessment and FACIT-Fatigue scores

**Fig. 2:**
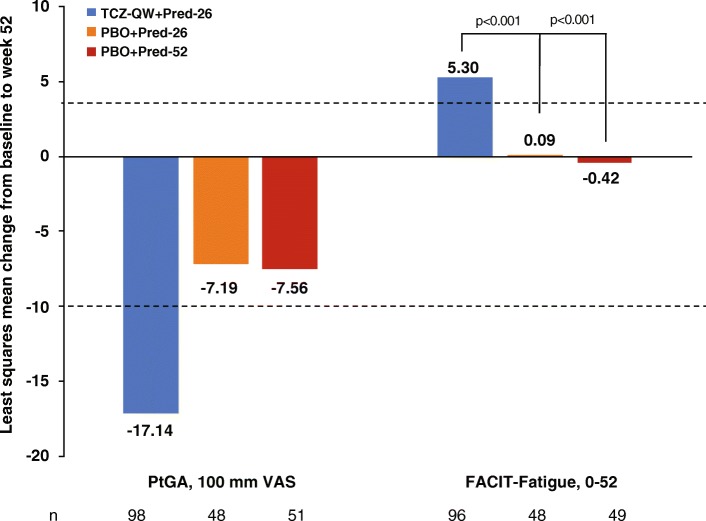
Change from baseline to week 52 in PtGA VAS and FACIT-Fatigue scores. Improvement is indicated by a negative change in PtGA and a positive change in FACIT-Fatigue score. *p < 0.001 vs TCZ-QW + Pred-26, upper dashed line = FACIT-Fatigue MCID, lower dashed line = PtGA MCID. *FACIT* Functional Assessment of Chronic Illness Therapy, *MCID* minimum clinically important difference, *PBO* placebo, *Pred-26* 26-week prednisone taper, *Pred-52* 52-week prednisone taper, *PtGA* Patient Global Assessment of Disease Activity, *QW* every week, *TCZ* tocilizumab

Reported improvements in LSM PtGA scores from baseline to week 52 with TCZ-QW + Pred-26 treatment were not statistically significantly different from those of either PBO + Pred group. LSM changes exceeded the MCID [19] only in the TCZ-QW + Pred-26 group (Fig. 2).

LSM increases in FACIT-Fatigue scores from baseline to week 52 in the TCZ-QW + Pred-26 group were significantly greater than in both PBO + Pred groups (*p* < 0.001) and exceeded the MCID [24] (Fig. 2).

### Patients reporting clinically meaningful improvements

**Fig. 3:**
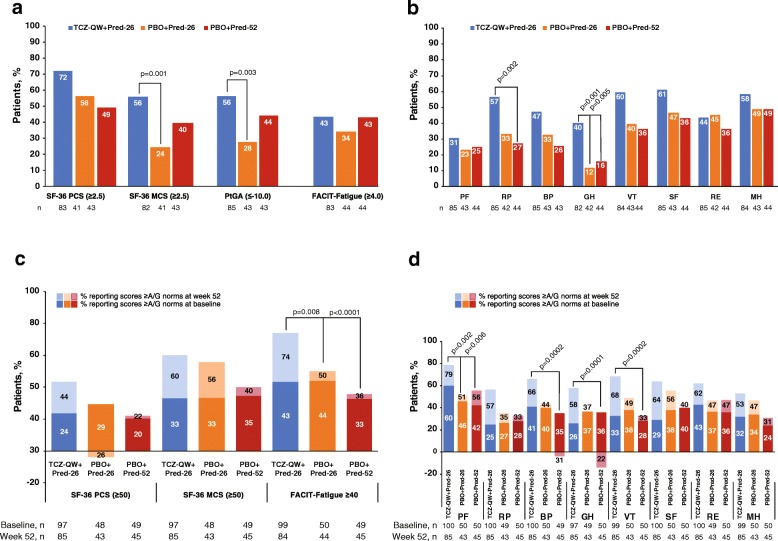
Proportions of patients reporting changes from baseline to week 52 meeting or exceeding MCID. MCIDs are based on patients with RA and SLE, and data are shown for PROs (**a**) and SF-36 domain scores (**b**) and proportions of patients reporting scores meeting or exceeding A/G-matched normative values in PROs (**c**) and SF-36 domain scores (**d**) at week 52. *A/G* age/gender, *BL* baseline, *BP* bodily pain, *FACIT* Functional Assessment of Chronic Illness Therapy, *GH* general health, *MCID* minimal clinically important difference, *MCS* Mental Component Summary, MH mental health, *PBO* placebo, *PCS* Physical Component Summary, *PF* physical function, *Pred-26* 26-week prednisone taper, *Pred-52* 52-week prednisone taper, *PROs* patient-reported outcomes, *QW* every week, *RA* rheumatoid arthritis, *RE* role emotional, *RP* role physical, *SF* social function, *SF-36* 36-Item Short-Form Health Survey, *SLE* systemic lupus erythematosus, *TCZ* tocilizumab, *VT* vitality

The proportions of patients who reported improvements that met or exceeded the MCID from baseline to week 52 in SF-36 PCS and MCS, PtGA and FACIT-Fatigue scores ranged from 43 to 72% in the TCZ-QW + Pred-26 group and were numerically higher than in either PBO + Pred group with the exception of PBO + Pred-52 for FACIT-Fatigue (Fig. 3a). Statistically significant differences in the percentages of patients reporting clinically meaningful improvements in PtGA (*p* = 0.0029) and SF-36 MCS (*p* = 0.0012) scores were observed in the TCZ-QW + Pred-26 group compared with the PBO + Pred-26 group, resulting in numbers needed to treat (NNTs) of 3.5 and 3.15, respectively. NNTs represent the number of patients who required treatment with an intervention, in this case TCZ-QW + Pred-26, to effect a clinically meaningful improvement in HRQOL (met or exceeded MCID) for one patient. Across SF-36 domain scores, 31 to 61% of patients receiving TCZ-QW + Pred-26 reported improvements that met or exceeded the MCID compared with 12 to 49% receiving PBO + Pred-26. The difference was statistically significant in the general health domain (40 vs 12%, *p* = 0.0010, NNT = 3.53) (Fig. 3b).

The proportions of patients reporting clinically meaningful improvements in SF-36 domain scores were numerically higher with TCZ-QW + Pred-26 than with PBO + Pred-52 (Fig. 3b). These differences were statistically significant in role physical (57 vs 27%, *p* = 0.0016, NNT = 3.42) and general health (40 vs 16%, *p* = 0.0053, NNT = 4.11) domains.

### Comparisons with age- and gender-matched normative values

Impaired HRQOL at baseline compared with A/G norms was evident across SF-36 PCS, MCS, domain and FACIT-Fatigue scores across all treatment groups, and few patients in any treatment group reported baseline scores that met or exceeded A/G norms across these PROs (Table 3, Figs. 1 and 3c, d).

The proportions of patients reporting scores that met or exceeded A/G norms in SF-36 PCS, MCS and FACIT-Fatigue increased from baseline to week 52 in the TCZ-QW + Pred-26 group, with generally smaller improvements in both PBO + Pred groups (Fig. 3c). The TCZ-QW + Pred-26 group demonstrated statistically significant differences in the proportions of patients reporting scores that met or exceeded A/G norms compared with the PBO + Pred-26 group in FACIT-Fatigue score (*p* = 0.008, Fig. 3c). Week 52 domain scores exceeded A/G norms in five of eight domains with TCZ-QW + Pred-26 (physical function, role physical, bodily pain, vitality and social function; Fig. 1) and met A/G norms in the remaining three domains. The proportions of patients reporting SF-36 physical function domain scores that met or exceeded A/G norms was significantly higher in the TCZ-QW + Pred-26 group than in the PBO + Pred-26 group (*p* = 0.002). The proportions of patients reporting bodily pain, general health, vitality, social function and mental health domain scores that met or exceeded A/G norms were lowest in the PBO + Pred-52 group. The patients in the PBO + Pred-52 group actually reported deterioration in the bodily pain and general health domains at week 52 (Fig. 3d).

## Discussion

The set of analyses reported here represents the most detailed investigation to date of fatigue and HRQOL in patients with GCA and the first report of such data from an RCT. Patients with GCA in all treatment groups reported lower FACIT-Fatigue, SF-36 PCS, MCS and domain scores compared with A/G norms at baseline, highlighting the impact of GCA itself on HRQOL. However, weekly TCZ treatment resulted in substantial and clinically meaningful decreases in patients’ fatigue and improvements in their HRQOL. Patients receiving TCZ-QW + Pred-26 reported statistically significantly greater improvements in SF-36 PCS, MCS, domain and FACIT-Fatigue scores than those in the PBO + Pred-26 and PBO + Pred-52 groups. Even more striking was the finding that improvements in the TCZ-QW + Pred-26 group met or exceeded A/G norms across every SF-36 domain. These findings are unprecedented in other HRQOL analyses across rheumatic diseases and highlight the importance of IL-6 in the underlying pathophysiology of GCA.

Data from these analyses underscore important differences between the two TCZ regimens used in this RCT. Patients treated with TCZ-QW + Pred-26 reported consistent and unequivocal improvements in HRQOL measures compared with patients in the PBO + Pred-26 and PBO + Pred-52 groups. In contrast, such improvements were not evident among patients treated with the every-other-week TCZ regimen (i.e., TCZ-Q2W + Pred-26). Thus, although both every week and every-other-week-TCZ treatment demonstrated clinical efficacy with regard to sustained GCA remission, the differences in HRQOL and fatigue observed between these two TCZ regimens argue in favour of weekly TCZ treatment.

Significant improvements in FACIT-Fatigue scores exceeding A/G norms were also reported for the TCZ-QW + Pred-26 group compared with both PBO + Pred groups. There also appeared to be a difference between the TCZ-QW + Pred-26 group and both PBO + Pred groups in PtGA scores, though these differences did not achieve statistical significance. One potential explanation for this is that patients who received prednisone escape therapy (i.e., had higher cumulative prednisone doses) were included in the current analyses. Glucocorticoids have the potential to make patients feel better by alleviating some disease symptoms without necessarily improving HRQOL or fatigue. In fact, patients in the PBO + Pred-52 group reported deterioration in SF-36 bodily pain and general health domains, a finding likely due to the relative excess of cumulative prednisone use in this group. PRO improvements reported in the TCZ-QW + Pred-26 group might have resulted not only from the effects of TCZ treatment on the symptoms of GCA but also from the lower cumulative glucocorticoid exposures enabled by TCZ treatment.

The observation that patients in the TCZ-QW + Pred-26 group reported improvements in HRQOL to levels exceeding A/G norms deserves further comment. A substantial body of research in recent years indicates that IL-6 is a crucial mediator not only of inflammation but also of pain, fatigue and even mood [26]. For example, injection of IL-6 into healthy persons is known to induce lowered moods [27]. The mechanism whereby IL-6 may mediate such changes is unclear, but one purported mechanism is through activation of the hypothalamic-pituitary axis [28]. Our findings support the concept that one mechanism whereby IL-6 inhibition improves HRQOL is through an elevating effect on mood.

## Conclusions

The addition of weekly TCZ to a regimen of glucocorticoids led to substantial and clinically meaningful improvements in fatigue and HRQOL. At 52 weeks, patients receiving weekly TCZ treatment reported fatigue and HRQOL scores that met or exceeded the value for A/G-matched norms.

## Abbreviations

A/G: Age/gender
BL: Baseline
BP: Bodily pain
ESR: Erythrocyte sedimentation rate
FACIT: Functional Assessment of Chronic Illness Therapy
GCA: Giant cell arteritis
GH: General health
HRQOL: Health-related quality of life
IL-6: Interleukin-6
IL-6Rα: Interleukin-6 receptor alpha
ITT: Intent-to-treat
LSM: Least squares mean
MCID: Minimal clinically important difference
MCS: Mental Component Summary
MH: Mental health
MID: Minimally important difference
NNT: Number needed to treat
NS: Not significant (*p* > 0.01)
PBO: Placebo
PCS: Physical Component Summary
PF: Physical function
PMR: Polymyalgia rheumatica
Pred-26: 26-week prednisone taper
Pred-52: 52-week prednisone taper
PRO: Patient-reported outcome
PtGA: Patient Global Assessment of Disease Activity
Q2W: Every 2 weeks
QW: Every week
RA: Rheumatoid arthritis
RCT: Randomised controlled trial
RE: Role emotional
RP: Role physical
SD: Standard deviation
SF: Social function
SF-36: 36-Item Short-Form Health Survey
SLE: Systemic lupus erythematosus
TCZ: Tocilizumab
VAS: Visual analogue scale
VT: Vitality
wk: Week
